# FBP1 knockdown decreases ovarian cancer formation and cisplatin resistance through EZH2-mediated H3K27me3

**DOI:** 10.1042/BSR20221002

**Published:** 2022-09-07

**Authors:** Xifeng Xiong, Xudong Lai, Jinli Zhang, Qingqi Meng, Pengzhen Wang, Shengnan Qin, Wei Liu, Yongxuan Wang, Zhuo Yao, Di Wang, Xiaojian Li, Zhihe Liu, Haixiong Miao

**Affiliations:** 1 Guangzhou Institute of Traumatic Surgery, Guangzhou Red Cross Hospital of Jinan University, Guangzhou 510220, China; 2 Department of Infectious Diseases, Guangzhou Red Cross Hospital of Jinan University, Guangzhou 510220, China; 3 Department of Breast Surgery, Guangzhou Red Cross Hospital of Jinan University, Guangzhou 510220, China; 4 Department of Pathology, Guangzhou Red Cross Hospital of Jinan University, Guangzhou 510220, China; 5 School of Life Sciences and biopharmaceutics, Guangdong Pharmaceutical University, Panyu District, Guangzhou 510006, China; 6 Department of Rehabilitation Medicine, The Second Affiliated Hospital of Guangzhou Medical University, 250 Changgang East Road, Guangzhou 510260, China; 7 Department of Burn and Plastic Surgery, Guangzhou Red Cross Hospital of Jinan University, Guangzhou 510220, China; 8 Department of Orthopedic Surgery, Guangzhou Red Cross Hospital of Jinan University, Guangzhou 510220, China

**Keywords:** cisplatin resistance, clinical significance, EZH2, FBP1, H3K27me3, ovarian cancer

## Abstract

Worldwide, ovarian cancer (OC) is the seventh common cancer and the second most common cause of cancer death in women. Due to high rates of relapse, there is an urgent need for the identification of new targets for OC treatment. The far-upstream element binding protein 1 (FBP1) and enhancer of zeste homolog 2 (EZH2) are emerging proto-oncogenes that regulate cell proliferation and metastasis. In the present study, Oncomine data analysis demonstrated that FBP1 was closely associated with the development of OC, and The Cancer Genome Atlas (TCGA) data analysis indicated that there was a positive correlation between FBP1 and EZH2 in ovarian tissues. Moreover, we found that FBP1 knockdown suppressed tumor formation in nude mice and cisplatin resistance of OC cells, but the role of FBP1 in the cisplatin resistance of OC cells remained unclear. In addition, we verified physical binding between FBP1 and EZH2 in OC cells, and we demonstrated that FBP1 knockdown enhanced cisplatin cytotoxicity in OC cells and down-regulated EZH2 expression and trimethylation of H3K27. These results suggested that FBP1 increases cisplatin resistance of OC cells by up-regulating EZH2/H3K27me3. Thus, FBP1 is a prospective novel target for the development of OC treatment.

## Introduction

Tumor development is a multistep and multistage complex biological process regulated by several signals, such as the Wnt signaling pathway and the TGF-β signaling pathway, as well as factors, including tumor suppressor, apoptosis, cell cycle, epigenetic alteration and drug resistance genes [1–7]. Ovarian cancer (OC) is the second most common cause of cancer in Occident and the third most common cause in Asia [8]. Worldwide, OC is the seventh common cancer and the second most common cause of cancer death in women [9]. The conventional treatment for OC is platinum-dependent cytotoxic chemotherapy after surgical operation [10], and treatment with surgery and chemotherapy is clinically effective for 50–80% of patients [11,12]. The combination therapy used for the treatment of OC consists of a platinum compound, either cisplatin or carboplatin, and a taxane, such as paclitaxel or docetaxel. In the last 30 years, the 5-year survival rate of OC has remained the same, while the 5-year survival rate of all cancers has increased by approximately 20% [13]. Even if OC patients gain benefits from platinum–paclitaxel combination therapy in the early phases of treatment, most of these patients relapse after an average of 18 months of survival without progression [14]. Therefore, there is an urgent need to improveresponse rates and to identify new targets for developing novel therapies for OC [15].

The occurrence and development of OC involve multiple factors, including tumor suppressor gene inactivation, oncogene activation and epigenetic abnormality. As a key regulator of the c-Myc proto-oncogene, far upstream element binding protein 1 (FBP1) plays an important role in cell proliferation [16,17]. In the past several years, several studies have shown that FBP1 is a novel proto-oncogene that has important functions in tumorigenesis [17]. The expression of FBP1 is low in most normal cells but is significantly increased in a variety of tumor cells, including liver cancer cells [18,19], oligodendroglioma [20], non-small cell lung cancer [21], breast cancer [22] and clear cell renal cancer [23]. In addition to activating c-Myc, FBP1 promotes cell proliferation by decreasing p21 [19] and promotes cell migration by increasing stathmin [18,21]. FBP1 also activates the replication of Hepatitis C virus [24–26] and Enterovirus 71 [27,28]. We previously reported that FBP1 is overexpressed in OC and inhibits the cell cycle and metastasis of OC cells [29,30].

Epigenetic polycomb group (PcG) proteins are transcriptional regulators in the form of polycomb repressor complexes (PRCs), and they play important roles in tumor development. Human PRCs are mainly divided into PRC1 and PRC2 [31]. PRC2 is conserved and is an important chromatin modifier. As the subunits of PRC2, EZH2, EED and SUZ12 are all indispensable for the methyltransferase activity of PRC2. PRC2 is the only identified methyltransferase that can catalyze mono-, di- and tri- methylation of histone H3 at lysine 27 (H3K27) [32,33]. H3K27me2 is abundant (accounting for approximately 50–70% of H3K27) and covers inter- and intragenic regions. H3K27me2 prevents inappropriate promoter or enhancer activities. H3K27me3 (accounting for 5–10% of H3K27) overlaps at the sites that PRC2 binds [33]. Most studies on PRC2 have been focused on H3K27me3, which is considered as a marker of PRC2-mediated gene repression [32,33]. By tri-methylating histone H3 (H3K27) at lysine 27 to form trimethylation histone H3 (H3K27Me3), EZH2 plays a key role in epigenetic gene regulation [34,35]. After trimethylation of H3K27, PRC2 regulates downstream genes by binding to specific gene sites, leading to tumorigenesis and multidirectional differentiation of stem cells [36]. EZH2 activates or inhibits the expression of downstream genes in a PRC2-dependent/-independent manner in different types of tumor cells [37–39], and it is involved in a variety of biological processes, including cell proliferation and apoptosis [40]. It has been reported that EZH2 is abnormally expressed in various tumors [41–43], including OC [29,30]. Moreover, EZH2 increases the resistance of OC to cisplatin [44,45]. In osteosarcoma cells, we demonstrated that FBP1 physically binds with EZH2 and that a positive mutual regulatory mechanism exists between FBP1 and EZH2 [46,47].

In the present study, we comprehensively analyzed the expression of FBP1 in OC described in three datasets, including Oncomine, Gene Expression Omnibus (GEO) and The Cancer Genome Atlas (TCGA), as well as its correlation with clinical features. Using Gene Set Enrichment Analysis (GSEA), we predicted the involvement of FBP1 in epigenetic regulation, histone methylation and DNA methylation. Furthermore, we analyzed the relationship between FBP1 and EZH2 in OC based on TCGA cohorts, and we confirmed a positive correlation between FBP1 and EZH2 in osteosarcoma cells. Because EZH2 is associated with OC resistance to cisplatin, we investigated the effects of FBP1 in cisplatin resistance of OC cells as well as the molecular mechanisms underlyingthis effect.

## Materials and methods

### Data collection and bioinformatics analysis

The clinical implication of FBP1 was determined based on the Oncomine dataset (https://www.oncomine.org). OC microarray data were downloaded from the GEO database (https://www.ncbi.nlm.nih.gov/geo/) comprises GSE12470 [48] (Platform: GPL887), GSE26712 [49] (Platform: GPL96), GSE93793 [50] (Platform: GPL17077) and GSE23554 [51] (Platform: GPL96). FBP1 mRNA expression and clinical data of OC were obtained from The Cancer Genome Atlas (TCGA) database (https://tcga-data.nci.nih.gov/tcga/) [52]. The relationship between FBP1 and EZH2 in ovarian tissues was analyzed using data from TCGA. The two-gene correlation map was generated using the ggstatsplot package in R.

### GSEA

In the present study, we obtained a list of all genes related to FBP1expression from TCGA. All genes were input into GSEA (https://www.gsea-msigdb.org/gsea/index.jsp), and the gene set permutations were performed 1000 times. The threshold values of adjusted *P*-value < 0.05 and FDR *q*-value < 0.25 were considered to be statistically significant.

### Samples collection

The experimental protocols of the present study were implemented after approval by the Medical Ethics Committee of Guangzhou Red Cross Hospital of Jinan University (Reference number: 2017-017-01). All methods were implemented in accordance with the Declaration of Helsinki. In total, 65 ovarian clinical samples were collected from our hospital. The normal clinical samples were received from patients who underwent adnexectomy for myoma or adenomyosis. The average age of patients was 48.8 years old (ranging from 17 to 73 years old). All OC patients were pathologically confirmed and did not receive preoperative chemotherapy, radiotherapy and/or immunotherapy. The samples were assigned into three groups, namely normal (20 samples), benign (25 samples) and malignant (20 samples). All tissues were fixed with 10% formalin and then embedded in paraffin.

### Immunohistochemical (IHC) staining

Immunohistochemical (IHC) was performed as previously reported [30]. Formalin-fixed paraffin-embedded ovarian tissue sections (5 μm) were deparaffinized (100% turpentine oil) and hydrated consecutively (100% ethanol, 95% ethanol, 90% ethanol, 85% ethanol, 75% ethanol and distilled water). The sections were incubated with citrate buffer (pH 6.0) for antigen retrieval using a microwave. Endogenous peroxidase activity was quenched using 3% hydrogen peroxide (H_2_O_2_), and the sections were blocked with 10% bovine serum albumin (BSA) for 30 min before incubation with FBP1, EZH2 and H3K27me3 antibodies overnight at 4°C. The sections were then incubated with horseradish peroxidase (HRP)-conjugated secondary antibody for 1 h at room temperature. After washing with phosphate-buffered saline (PBS), the reaction was visualized by incubating the sections with 3,3′-diaminobenzidine (DAB) followed by immersion in hematoxylin for 5 min. Finally, the sections were dehydrated with graded alcohol and sealed. Negative control sections were incubated with blocking reagent alone in the absence of primary antibody.

Images were acquired using a 40× objective, and image processing and analyses were performed using Image-Pro Plus 6.0 software (Media Cybernetics, Shanghai, China). The intensity of the immunohistochemical reaction was expressed as integral optical density (IOD) of DAB brown reaction products. The results of five separate measurements for each sample are expressed as the mean ± standard deviation (SD) [30].

### Antibodies and reagents

FBP1 (Cat. No. ab213525) antibody was obtained from Abcam (Cambridge, England, U.K.), and the EZH2 (Cat. No. 5246), H3K27me3 (Cat. No. 9733) and GAPDH (Cat. No. 5174) antibodies were purchased from Cell Signaling Technology (Danvers, MA, U.S.A.). Dulbecco’s modified Eagle medium (DMEM), FBS and L-glutamine were obtained from Gibco (Gaithersburg, MD, U.S.A.). Penicillin and streptomycin sulfate were purchased from Hyclone (Logan, Utah, U.S.A.). The CellTiter 96 AQueous One Solution Reagent (MTS) was purchased from Promega (Madison, WI, U.S.A.). Cisplatin was purchased from QiLu Pharmaceutical (Shandong, China).

### Cell culture and cell viability assay

As previously reported [29], human OC SKOV-3 cells were cultured in DMEM with 10% (v/v) FBS, 2 mM L-glutamine, 100 U/ml penicillin and 100 μg/ml streptomycin at 37°C and 5% CO_2_ in a humidified incubator. The medium was changed every 2–3 days, and cultures were passaged using 0.25% trypsin (Gibco, Gaithersburg, MD, U.S.A.).

A pSi-LVRH1GP lentiviral vector with a puromycin resistance cassette (GeneCopoeia, Rockville, MD, U.S.A.) was used to express a short hairpin (shRNA) to knockdown FBP1 expression [29]. The control vector expressed a scrambled sequence (5′-GCT TCG CGC CGT AGT CTT A-3′) and was named pSi-LV-FBP1-C. The FBP1 knockdown vector expressed aa 1671-1691 of FBP1(5′-GCA GGA ACG GAT CCA AAT TCA-3′) and was named pSi-LV-FBP1-KD. FBP1knockdown (FBP1-KD) and FBP1 control (FBP1-C) SKOV3 cells were generated as previously reported [29].

SKOV-3 cells were seeded into 96-well plates at a density of 5 × 10^3^ cells per well and incubated overnight. SKOV-3 cells were then treated with a series of cisplatin concentrations (0, 1, 5, 10, 15, 20, 30, 40 and 50 μM) for 48 h. For FBP1-C and FBP1-KD, SKOV-3 cells were seeded into a 96-well plate at a density of 5 × 10^3^ cells/well and incubated overnight. FBP1-C and FBP1-KD SKOV-3 cells were treated with 10 μM cisplatin for 48 h. Cell viability was measured using MTS in accordance with the manufacturer’s protocol (Promega, Madison, WI, U.S.A.), and the absorbance at awavelength of 490 nm was read in an automated plate reader (BioTek, Winooski, VT, U.S.A.).

### Cell apoptosis and caspase-3/7 activity assay

The apoptosis of FBP1-C and FBP1-KD SKOV-3 cells was assessed with an apoptosis detection kit (BD Biosciences, San Diego, CA, U.S.A.). Cells were incubated with V450 solution for 30 min in the dark followed by incubation with 7-AAD solution for 5 min. The percentage of apoptotic cells was detected and analyzed by FACScan Flow Cytometer (BD Biosciences) and BD CellQuest Pro software (BD Biosciences).

For the caspase-3/7 activity assay, FBP1-C and FBP1-KD SKOV-3 cells (5 × 10^3^ cells) were seeded into a 96-well plate and incubated overnight. FBP1-C and FBP1-KD SKOV-3 cells were cultured with 10 μM cisplatin for 48 h. The activities of caspase-3/7 were evaluated by the Caspase-Glo 3/7 assay kit (Promega). Following the manufacturer’s instructions, the Caspase-GloR 3/7 buffer and lyophilized Caspase-GloR 3/7 substrate were equilibrated to room temperature before use. Caspase-GloR 3/7 substrate was dissolved thoroughly in Caspase-GloR 3/7 buffer to form the Caspase-GloR 3/7 reagent. After equilibrating the 96-well plate containing treated cells to room temperature, 100 μl of Caspase-Glo 3/7 reagent was added to each well, and the plate was incubated at 22°C with gentle shaking. After 2 h, the luminescence was measured using an luminometer (Promega).

### Quantitative real-time polymerase chain reaction (qRT-PCR)

Total RNA was extracted from FBP1-KD and FBP1-C SKOV-3 cells using TRIzol Reagent (Invitrogen, Carlsbad, CA, U.S.A.) according to the manufacturer’s instructions [46]. After the quality and quantity of the extracted total RNA were confirmed by a spectrophotometer (Beckman, Berkeley, CA, U.S.A.), cDNA was synthesized using a reverse transcription kit (TaKaRa, Dalian, China) according to the manufacturer’s protocol. The following primers were used for qRT-PCR: EZH2 forward, 5′-ACCAGCATTTGGAGGGAGC-3′; EZH2 reverse, 5′-TGGGAAGCCGTCCTCTTCT-3′; FBP1 forward, 5′-TGATTCCAGCTAGCAAGGCA-3′; FBP1 reverse, 5′-CGGCCCGTCTTGAATCATAA-3′; GAPDH forward, 5′-GATTCCACCCATGGCAAATT-3′; and GAPDH reverse, 5′-TCTCGCTCCTGGAAGATGGT-3′. All reactions were performed on an Applied Biosystems 7300 PCR system (Applied Biosystems, Foster, CA, U.S.A.). A master mix was prepared on ice containing 1 µl of cDNA sample, 12.5 µl of SYBR Green Real-time PCR Master Mix (TaKaRa) and 1 µl of 10 µM primers. The final volume was then adjusted to 20 µl with water. Reactions were performed under the following cycling conditions: initial denaturation at 95°C for 3 min; and 40 cycles of denaturation at 95°C for 15 s, annealing at 60°C for 30 s and extension at 72°C for 45 s. Relative quantification was performed using the Ct (2^−△△Ct^) method. PCR amplification was performed in triplicate to verify the results.

### Western blot analysis

As previously reported [46], cells were lysed in modified RIPA buffer (150 mM NaCl, 1% NP-40, 50 mM Tris-Cl [pH 8.0] and 0.1% SDS) supplemented with PMSF (1 mM). After homogenization, the lysate was incubated on ice for 30 min and centrifuged at 12000 ***g*** for 15 min at 4°C. Protein concentrations were checked by a Bio-Rad protein assay (Bio-Rad, Hercules, CA, U.S.A.). Protein samples (50 µg) were resolved by 12% SDS-PAGE and transferred to PVDF membranes (Millipore, Bedford, MA, U.S.A.). According to the expected molecular weights of the proteins, the membranes were cut into narrow pieces. The membrane pieces were blocked with 5% nonfat milk for 1 h and then incubated with primary antibodies overnight at 4°C. After washing, membranes were incubated with HRP-labeled secondary antibodies for 1 h at room temperature and detected with ECL-Plus detection systems (Pierce, Rockford, IL, U.S.A.). The relative abundance was quantified by densitometry using Quantity One 4.6.7 software (Bio-Rad, Hercules, CA, U.S.A.).

### Co-immunoprecipitation (Co-IP) experiments

SKOV-3 cells were harvested and lysed in lysis buffer (150 mM NaCl, 50 mM Tris-HCl, 1 mM EDTA [pH 7.5] and 1% NP-40) containing protease inhibitor (Roche, CA, U.S.A.). Whole cell lysates were incubated with an anti-FBP1 antibody, anti-EZH2 antibody or isotype IgG (Abcam, Cambridge, U.K.) at 4°C for 2 h followed by incubation with prepared Protein A+G agarose beads (Santa Cruz, CA, U.S.A.) at 4°C overnight with rotation. After washing with lysis buffer, the precipitates were eluted in SDS-PAGE loading buffer by boiling for 5 min. The supernatants were then resolved by SDS-PAGE and transferred to PVDF membranes. Immunoblotting using appropriate antibodies was conducted using a standard Western blot protocol [47].

### Xenograft tumor model

For tumorigenicity examination, 5-week-old female nude athymic BALB/c mice were obtained from Guangdong Animal Experiment Center (Guangdong, China). The care and use of animals in this experiment was in accordance with the Regulations on the Administration of Laboratory Animals of Guangdong Province. The mice were acclimated to the environment for one week at the Institute of Laboratory Animal Science of Jinan University before tumor formation experiments. To evaluate the role of FBP1 in tumor formation, FBP1-KD or FBP1-C SKOV-3 cells (2 × 10^6^) were injected subcutaneously into the dorsal flanks of mice (four mice for FBP1-C cells and three mice for FBP1-KD cells). After 4 weeks of injection, mice were anesthetized and euthanized using 40 mg/kg ketamine (Sigma-Aldrich, U.S.A.) and 5 mg/kg xylazine (Sigma-Aldrich, U.S.A.), respectively. The tumors were collected, and the tumor weights were measured. Each tumor was divided into three portions for qRT-PCR, Western blot and IHC analyses. Ethics approval for the animal experiment was obtained from the Medical Ethics Committee of Guangzhou Red Cross Hospital of Jinan University (Reference number: 2017-017-01).

### Statistical analysis

R software (version. 3.6.2; http://www.Rproject.org) was used for the bioinformatics analyses. The difference between FBP1 expression in normal and tumor tissues was analyzed by the Wilcoxon rank-sum test. The association between clinical characteristics and FBP1 expression was evaluatedby logistic regression, Kruskal–Wallis test, Wilcoxon signed-rank test, Fisher exact test or Chi-square test. In all tests, *P*<0.05 was considered statistically significant.

For experiment data analysis, all data were obtained from at least three independent experiments. Data were analyzed using an independent *t*-test and one-way analysis of variance (ANOVA), and data are presented as the mean ± SD. Spearman’s correlation analysis was used to evaluate the correlation between quantitative variables without a normal distribution. *P*<0.05 was considered statistically significant.

## Results

### Association between FPB1 expression and clinical characteristics

We analyzed FBP1 expression in 379 OC patients from TCGA database. By Fisher’s exact test or Chi-square test, we found that there was a significant correlation between FBP1 expression and FIGO stage (*P*=0.006), but there was no significant correlation with other clinical characteristics, including lymphatic invasion, primary therapy outcome, age, histological grade, anatomic neoplasm subdivision, venous invasion, tumor residual and tumor status (Table 1). The low or high FBP1 expression is just distinguished according to TPM in Table 1. The median expression of FBP1 from TCGA database is believed as TPM and the TPM is 49.16. It is believed as high expression if TPM is more than 49.16 and low expression if TPM is less than 49.16.

**Table 1:** Association between FBP1 expression and clinicopathologic features in ovarian cancer samples from the TCGA database

Characteristic	FBP1 low expression	FBP1 high expression	*P*
*n* (%)	189 (50%)	190 (50%)	
FIGO stage			0.006
Stage I	0 (0%)	1 (0.3%)	
Stage II	5 (1.3%)	18 (4.7%)	
Stage III	147 (38.9%)	148 (38.9%)	
Stage IV	35 (9%)	22 (5.8%)	
Deficiency	2 (0.5%)	1 (0.3%)	
Lymphatic invasion			0.100
No	18 (4.7%)	30 (7.9%)	
Yes	54 (14.3%)	47 (12.4%)	
Deficiency	117 (31.0%)	113 (29.7%)	
Primary therapy outcome			0.945
PD	15 (4.0%)	12 (3.2%)	
SD	11 (2.9%)	11 (2.9%)	
PR	21 (5.6%)	22 (5.8%)	
CR	107 (28.3%)	109 (28.7%)	
Deficiency	35 (9.2%)	36 (9.4%)	
Age			0.646
≤60	101 (26.7%)	107 (28.2%)	
>60	88 (23.3%)	83 (21.8%)	
Histologic grade			0.142
G1	1 (0.3%)	0 (0%)	
G2	27 (7.1%)	18 (4.7%)	
G3	155 (41.0%)	167 (43.9%)	
G4	0 (0%)	1 (0.3%)	
Deficiency	6 (1.6%)	4 (1.1%)	
Anatomic neoplasm subdivision			0.750
Unilateral	53 (14.0%)	49 (12.9%)	
Bilateral	126 (33.3%)	129 (33.9%)	
Deficiency	10 (2.7%)	12 (3.2%)	
Venous invasion			0.321
No	15 (4%)	26 (6.8%)	
Yes	31 (8.2%)	33 (8.7%)	
Deficiency	143 (37.8%)	131 (34.5%)	
Tumor residual			0.085
NRD	28 (7.4%)	39 (10.3%)	
RD	146 (38.6%)	122 (32.1%)	
Deficiency	15 (4%)	29 (7.6%)	
Tumor status			0.113
Tumor free	29 (7.7%)	43 (11.3%)	
With tumor	137(36.2%)	128 (33.7%)	
Deficiency	23 (6.1%)	19 (5%)	
Age, meidan (IQR)	60 (51, 70)	58 (51, 67)	0.365

Abbreviations: CR, complete remission; IQR, interquartile range; NRD, no residual disease; PD, progressive disease; PR, partial remission; RD, residual disease; SD, stable disease.

**Figure 1:**
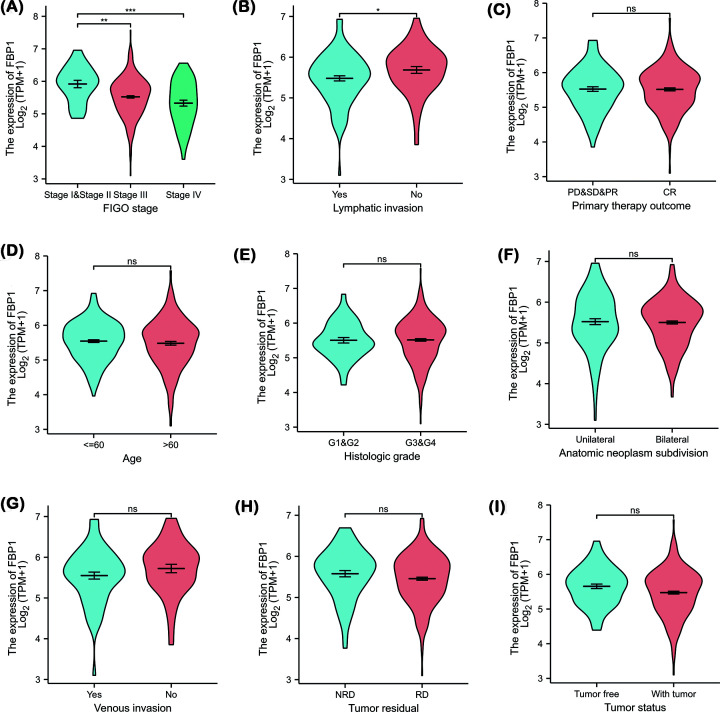
Association between FBP1 expression and clinicopathological characteristics in OC from TCGA database (**A**) FBP1 expression in FIGO stage (stage III vs. stages I and II, *P*<0.01; stage IV vs. stages I and II; *P*<0.001). (**B**) FBP1 expression in lymphatic invasion (yes vs. no; *P*<0.05). (**C**) FBP1 expression in primary therapy outcome (CR vs. PD, SD and PR; *P*>0.05). (**D**) FBP1 expression in age (≤60 vs. >60; *P*>0.05). (**E**) FBP1 expression in histologic grade (G3 and G4 vs. G1 and G2; *P*>0.05). (**F**) FBP1 expression in anatomic neoplasm subdivision (bilateral vs. unilateral; *P*>0.05). (**G**) FBP1 expression in venous invasion (yes vs. no; *P*>0.05). (**H**) FBP1 expression in tumor residual disease (RD vs. NRD; *P*>0.05). (**I**) FBP1 expression in cancer status (with tumor vs. tumor free; *P*>0.05).

We also used Wilcoxon signed-rank test and Kruskal–Wallis test to analyze the association between FBP1 expression and clinical characteristics. Regarding the FIGO stage, lymphatic invasion showed a significant correlation with FBP1 expression (Figure 1A and B). FBP1 expression was significantly lower in FIGO stage III (*P*<0.01) and IV (*P*<0.001) than in FIGO stages I and II, and FBP1 expression was significantly lower in lymphatic invasion OC than in lymphatic non-invasion OC. There were no significant differences in FBP1 expression with regard to other clinical characteristics (Figure 1C–I).

Furthermore, we used univariate logistic regression to analyze the relationship between FBP1 expression and clinicopathological factors of OC. In addition to FIGO stage (OR = 0.246, 95% CI: 0.080–0.627, *P*=0.006), tumor residual (OR = 0.564, 95% CI: 0.325–0.967, *P*=0.039) also showed a significant correlation with FBP1 expression (Table 2). There was no significant correlation of FBP1 expression with the other clinical characteristics (Table 2).

**Table 2:** The relationship between the clinicopathological factors of ovarian cancer and FBP1 expression by using logistic analysis

Characteristics	Total (*n*)	Odds ratio (OR)	*P*
FIGO stage (stage I and II vs. stage III and IV)	376	0.246 (0.080–0.627)	**0.006**
Primary therapy outcome (PD and SD and PR vs. CR)	308	1.437 (0.880–2.360)	0.149
Age (≤60 vs. >60)	379	0.853 (0.569–1.279)	0.442
Histologic grade (G1 and 2 vs. G3 and 4)	369	1.243 (0.670–2.329)	0.491
Anatomic neoplasm subdivision (unilateral vs. bilateral)	357	1.065 (0.672–1.687)	0.789
Venous invasion (no vs. yes)	105	0.577 (0.255–1.277)	0.179
Lymphatic invasion (no vs. yes)	149	0.517 (0.251–1.040)	0.068
Tumor residual (NRD vs. RD)	335	0.564 (0.325–0.967)	**0.039**
Tumor status (tumor free vs. with tumor)	337	0.602 (0.352–1.018)	0.060

Abbreviations: CR, complete remission; NRD, no residual disease; PD, progressive disease; PR, partial remission; RD, residual disease; SD, stable disease.

These statistical analyses demonstrated that FBP1 is associated with FIGO stage.

### FBP1 expression in ovarian tissues

**Figure 2:**
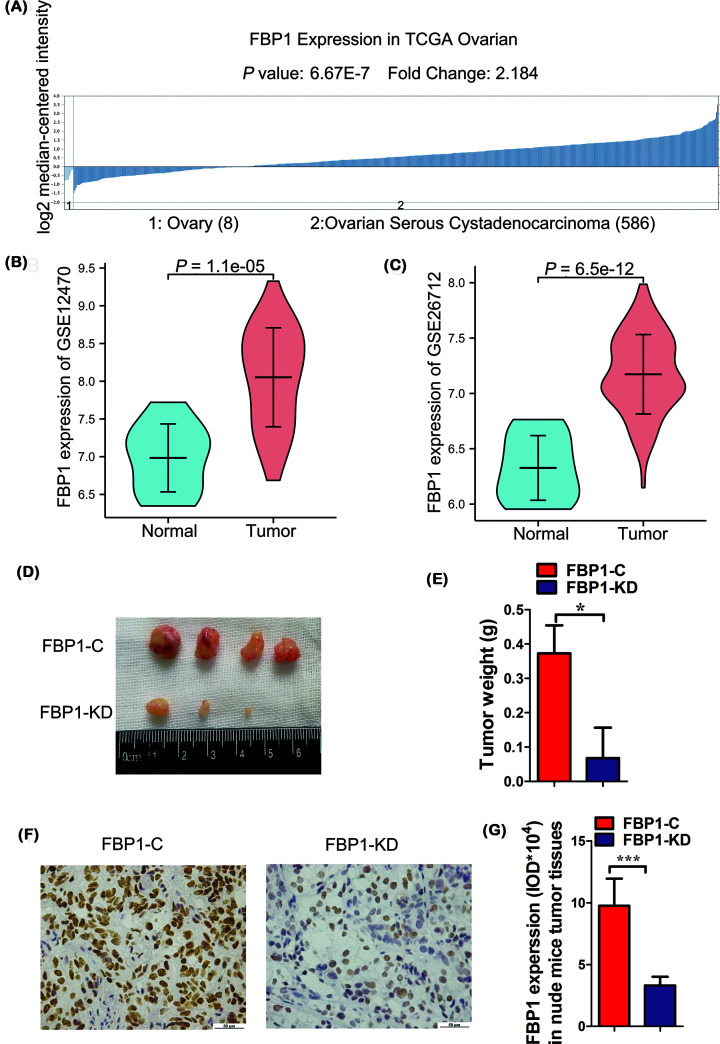
FBP1 promotes OC cell proliferation and tumor formation (**A**) FBP1 expression was closely associated with OC development based on Oncomine datasets. (**B**) FBP1 expression in normal and OC tissues in the GSE12470 dataset. (**C**) FBP1 expression in normal and OC tissues in the GSE26712 dataset. (**D**) Tumor formation in nude mice following the injection of FBP1-C or FBP1-KD SKOV-3 cells. (**E**) Tumor weight after the injection of FBP1-C or FBP1-KD SKOV-3 cells. (**F,G**) FBP1 expression in xenograft tumor tissues; magnification = 400; bar, 50 μm; *P<0.05, ***P<0.001.

Because FBP1 is the promoter of the c-Myc oncogene, we investigated the Oncomine database (https://www.oncomine.org), which demonstrated that FBP1 expression increased with the development of carcinoma. The log2 median-centered intensity of adenocarcinoma was 2.184-fold greater than that of normal ovarian tissues (*P* = 6.67E-7, Figure 2A). In the GSE12470 and GSE26712 datasets, FBP1 was overexpressed in OC (Figure 2B,C). These data demonstrated that the expression of FBP1 is higher in ovarian tumor tissue than in normal ovarian tissue.

### FBP1 promotes OC cell proliferation and cancer formation *in vivo*

To investigate the function of FBP1 in tumorigenesis, we performed a nude mouse xenograft experiment. SKOV-3 FBP1-KD cells or FBP1-C cells were subcutaneously inoculated into the dorsal flank of nude mice. After injection, tumors were present in all mice in the fourth week. After the tumors were removed, the tumor weights were measured. FBP1-KD tumors were smaller than FBP1-C tumors (Figure 2D,E). The expression of FBP1 was significantly lower in FBP1-KD tumors than in FBP1-C tumors according to IHC staining (Figure 2F), and the DAB intensities (mean ± SD) were lower in FBP1-KD tumors than in FBP1-C tumors (Figure 2G). These data demonstrated that FBP1 promotes OC cell proliferation and tumor formation in vivo.

### FBP1 enhances cisplatin resistance of OC cells

**Figure 3:**
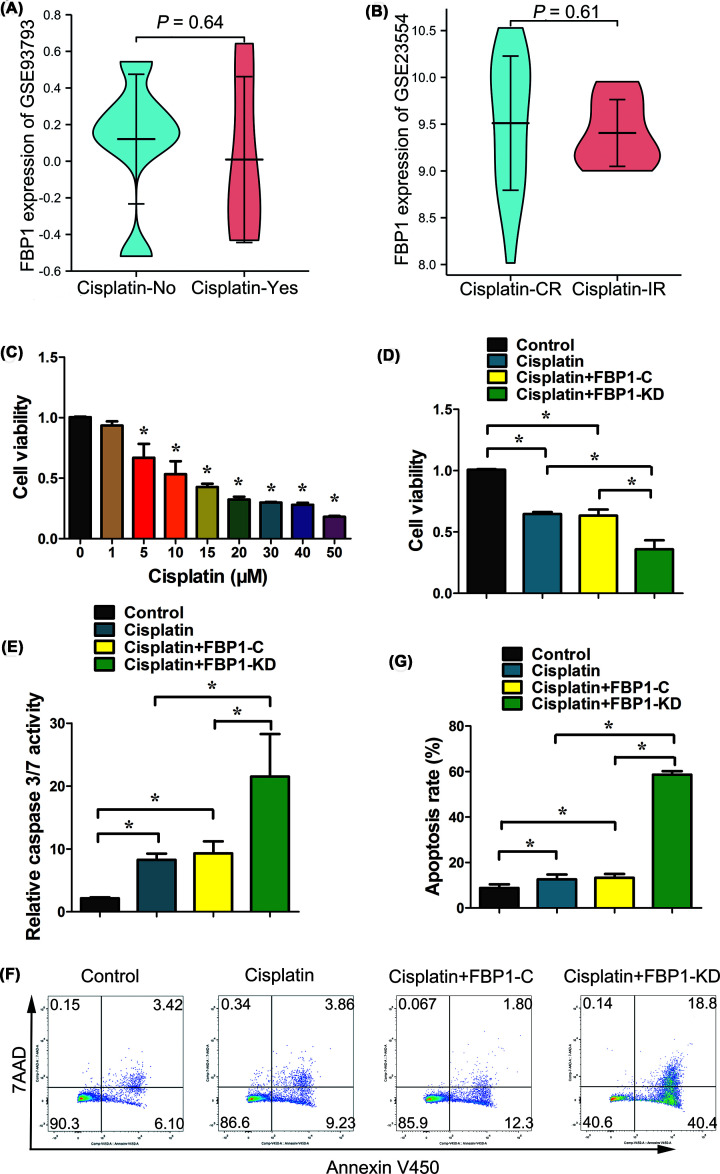
FBP1 enhances cisplatin resistance of OC cells (**A**) FBP1 expression in OC treated without or with cisplatin from the GSE93793 dataset. (**B)** FBP1 expression in cisplatin complete response (CR) or incomplete response (IC) OC from the GSE23554 dataset. (**C**) Cisplatin inhibited the viability of ovarian SKOV-3 cells. After treatment with indicated concentrations of cisplatin for 48 h, SKOV-3 cell viabilities were evaluated by the MTS method. (**D**) FBP1 knockdown enhanced cisplatin sensitivity of ovarian SKOV-3 cells. After treatment with 10 μM cisplatin for 48 h, FBP1-C and FBP1-KD SKOV-3 cell viabilities were evaluated by the MTS method. (**E**) FBP1 knockdown accelerated caspase-3/7 activities induced by cisplatin treatment. (**F,G**) FBP1 knockdown accelerated apoptosis (F) and apoptosis rate (G) as analyzed by flow cytometry analysis. Apoptosis rate was defined as the percentage of apoptotic cells; *P<0.05.

The platinum–paclitaxel regimen is the main treatment used for OC. Cisplatin and carboplatin are two agents used inthis combination regimen. Analysis of the GSE93793 cohort demonstrated that there was no difference in FBP1 expression in the OC patients who were treated with or without cisplatin (Figure 3A). Furthermore, we analyzed the expression of FBP1 in cisplatin complete response (CR) patients and incomplete response (IR) patients in the GSE24554 cohort, and we found that there was no difference in FBP1 expression levels between these two groups (Figure 3B). Herein, we evaluated cisplatin cytotoxicity in OC cells by MTS assay. Cells were treated with 1, 5, 10, 15, 20, 30, 40 and 50 μM cisplatin, and the viability of SKOV-3 cells was significantly inhibited at 5, 10, 15, 20, 30, 40 and 50 μM cisplatin in a dose-dependent manner (Figure 3C). To determine the role of FBP1 in the cisplatin resistance of OC cells, FBP1-C and FBP1-KD SKOV-3 cells were exposed to 10 μM cisplatin. The MTS assay revealed that FBP1 knockdown exacerbated the decrease of SKOV-3 cell viability induced by cisplatin (Figure 3D). Cisplatin treatment elevated caspase-3/7 activity in SKOV-3 cells, and the knockdown of FBP1 enhanced this effect (Figure 3E). As indicated by flow cytometryanalyses, cisplatin treatment resulted in a higher proportion of apoptotic cells, whileFBP1 knockdown significantly enhanced the proportion of apoptotic cells (Figure 3F,G). Taken together, these results indicated that FBP1knockdown decreases the cisplatin resistance of OC cells, which disagreed with the GEO database analysis results.

### Knockdown of FBP1 decreases EZH2 and H3K27me3 expression

**Figure 4:**
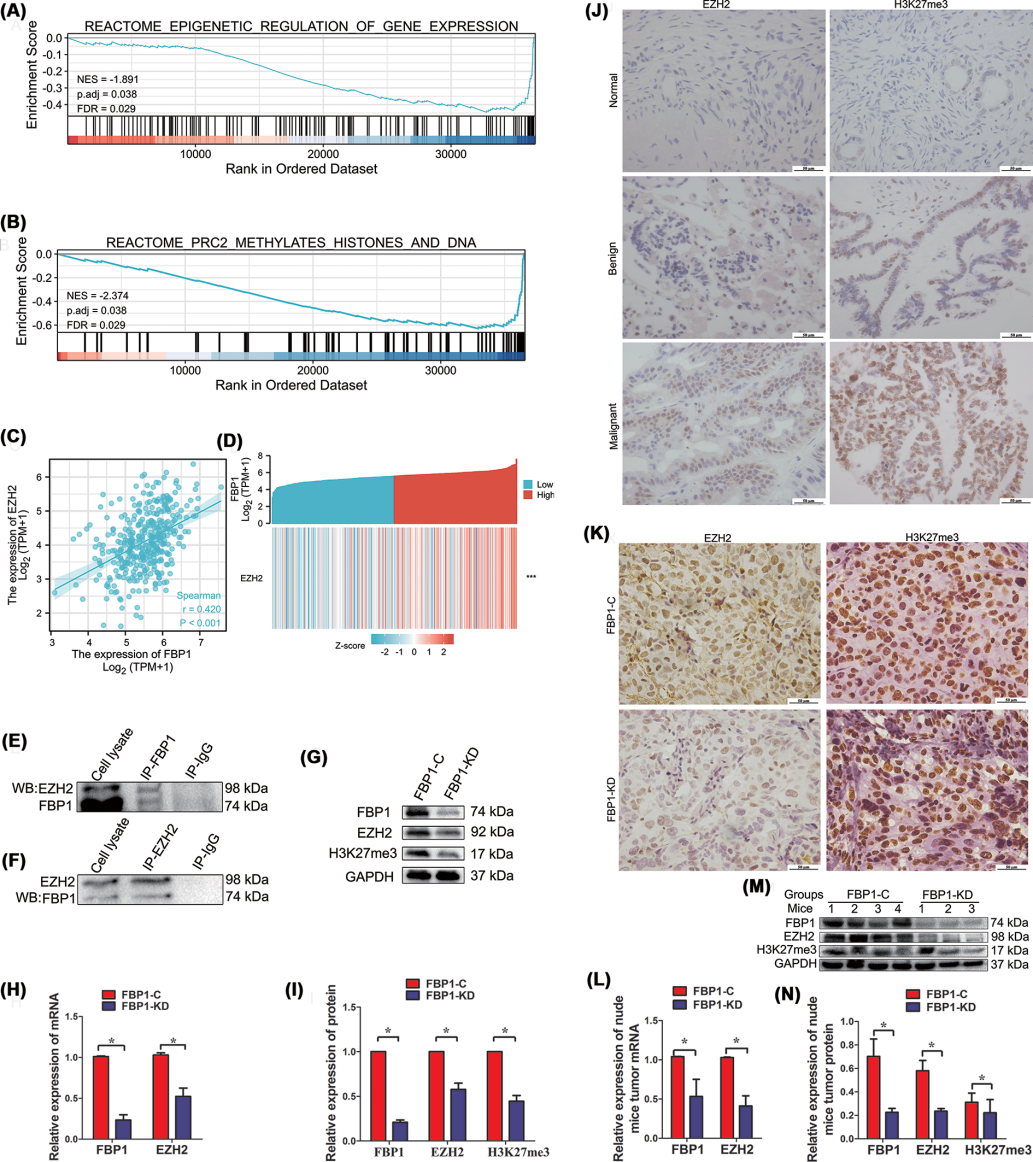
FBP1 promotes the EZH2/ H3K27me3 signaling pathway (**A**) FBP1 was differentially enriched in epigenetic regulation of gene expression according to GSEA. (**B**) FBP1 was differentially enriched in PRC2 methylation of histone and DNA according to GSEA. (**C**) The relationship between FBP1 and EZH2 was determined based on TCGA dataset. (**D**) Heatmap of co-expression of FBP1 and EZH2 based on TCGA dataset. (**E,F**) The physical interaction between FBP1 and EZH2 in OC SKOV-3 cellswasdetected by Co-IP assay using FBP1 or EZH2 antibody, respectively. (**G**) Protein expression of FBP1, EZH2 and H3K27me3 in FBP1-C and FBP1-KD OC SKOV-3 cells as detected by Western blot analysis. (**H**) mRNA expression levels of FBP1 and EZH2 in ovarian cells as detected by qRT-PCR. (**I)** Relative protein expression levels of FBP1, EZH2 and H3K27me3 in FBP1-C and FBP1-KD OC SKOV-3 cells. (**J**) Expression of EZH2 and H3K27me3 in normal, benign and malignant ovarian tissues as detected by IHC. (**K**) Expression of EZH2 and H3K27me3 in xenograft tumor tissues as detected by IHC. (**L**) mRNA expression of FBP1 and EZH2 in xenograft tumor tissues as detected by qRT-PCR. (**M**) Protein expression of FBP1, EZH2 and H3K27me3 in xenograft tumor tissues as detected by Western blot analysis. (**N)** Relative protein expression of FBP1, EZH2 and H3K27me3 in xenograft tumor tissues. Note: The blots were cropped from the same gel; magnification: ×400; *P<0.05. ***P<0.001; bar: 50 μm.

As a transcriptional regulator, PRC2 plays an important role in tumor development. We used GSEA to identify FBP1-related signaling pathways in OC. The results showed that the enrichment of FBP1 in OC was associated with epigenetic regulation of gene expression, histone methylation and DNA methylation promoted by PRC2 (Figure 4A,B and Table 3). Analysis of the FBP1 and EZH2 mRNA sequences from TCGA dataset confirmed a positive relationship between FBP1 and EZH2 in ovarian tissues (*r* = 0.42, Figure 4C). Similarly, the heatmap of co-expressed genes showed a consistent expression of EZH2 in response to FBP1 (Figure 4D). Thus, we hypothesized that FBP1 enhances cisplatin resistance of OC cells through promoting EZH2 and H3K27me3 expression. Co-IP experiments using FBP1 or EZH2 antibodies confirmed EZH2 or FBP1 in the corresponding reaction by Western blot, respectively. Co-IP experiments confirmed the interaction between FBP1 and EZH2 in OC SKOV-3 cells (Figure 4E,F). FBP1 knockdown significantly decreased EZH2 expression both at the protein and mRNA levels (Figure 4G–I). As one ofthe downstream targets of EZH2, the expression of H3K27me3 protein was also significantly down-regulated by FBP1 knockdown (Figure 4G,I).

**Table 3:** Results of gene set enrichment analysis (GSEA)

Description	Set size	Enrichment score	NES	Value	*P* adjust	FDR *q* value	Rank	Leading_edge
REACTOME_EPIGENETIC_REGULATION_OF_GENE_EXPRESSION	146	-0.44602192	-1.89149556	0.00123305	0.03822158	0.02879123	3812	tags = 21%, list = 10%, signal = 19%
REACTOME_PRC2_METHYLATES_HISTONES_AND_DNA	71	-0.62990528	-2.37378572	0.0013369	0.03822158	0.02879123	3654	tags = 39%, list = 10%, signal = 36%

Abbreviations: FDR, false discovery rate; NES, normalized enrichment score. Gene sets with *P* adjust < 0.05 and FDR *q* value < 0.05 are considered as significant.

To identify whether the expression of EZH2 and H3K27me3 is related with OC development, we measured the protein expression of EZH2 and H3K27me3 in ovarian tissues by IHC. As shown in Figure 4J, EZH2 was mainly localized in the nucleus of ovarian cells and increased as the OC developed. The expression of EZH2 in malignant cells was significantly higher than that in benign or normal tissues. Similarly, H3K27me3 expression in malignant cells was significantly higher than thatin benign or normal tissues (Figure 4J). According to IHC, the intensities of EZH2 and H3k27me3 were weaker in the xenograft tumors of the FBP1-KD group compared to the FBP1-C group (Figure 4K). Compared with the FBP1-C group, the expression of EZH2 was lower in the xenograft tumors of the FBP1-KD group at both mRNA and protein levels according to qRT-PCR and Western blot analyses, respectively (Figure 4L–N). Similarly, a significantly lower expression of H3K27me3 was identified in the FBP1-KD group compared with the FBP1-C group (Figure 4M,N). Based on these data, the promoting effect of FBP1 on ovarian tumor formation and cisplatin resistance may be attributed to the up-regulation of the EZH2/H3k27me3 signaling pathway.

## Discussion

FBP1 is a DNA- and RNA-binding protein that acts as apotent pro-proliferative and anti-apoptotic factor, and it is involved in diverse cellular processes [53]. Importantly, emerging studies have revealed that FBP1 plays oncogenic roles in multiple cancers [18,23,54,55]. In the present study, the Oncomine and TCGA results showed that FBP1 was highly expressed in OC tissues compared with normal tissues, which was consistent with our previous results in OC [29]. Additionally, FBP1 was highly expressed in OC in the GSE12470 and GSE26712 datasets. In addition, FBP1 knockout reduced tumor formation in transplanted nude mice *in vivo*, which indicated that FBP1 promoted OC development. Analysis of the association between FBP1 expression and the clinical characteristics of OC from TCGA database demonstrated that FBP1 was down-regulated in OC with a higher FIGO stage and lymphatic invasion OC. These results indicated that FBP1 may have an important effect on the pathogenesis and tumor formation of OC.

Chemotherapy resistance is a major obstacle to effective cancer treatment [56]. Despite improvements in chemotherapy regimens, most OC patients will eventually die of this disease due to increasing chemoresistance when the disease progresses [57]. In general, chemoresistance is classifiedinto innate and obtained resistance, eventhough it is difficult to make a distinction between these two mechanisms. Innate resistance can be prevented with mechanisms, such as lower usage of drugs [58], drug degradation [59], poor vascularization [60] and ECM-related environment resistance [61]. Obtained resistance comes from the adaptation of tumor cells to the environment through Darwinian selection, which can include the modulation of gene expression to increase cell viability and cell tolerability of genetic damage. Many molecules and signal pathways are involved in OC chemotherapy resistance. Cancerous inhibitor of protein phosphatase 2A (CIP2A) has been identified as a human oncoprotein that inhibits c-Myc protein degradation. Knockdown of CIP2A increases cisplatin sensitization of OC cells [62], and knockout of high-mobility group box 3 (HMGB3) protein attenuates cisplatin resistance in human OC cells [63]. In the present study, we analyzed FBP1 expression in the GSE93793 and GSE24554 cohort datasets, and we found that there was no difference in FBP1 expression between cisplatin-treated OC patients and non-cisplatin-treated OC patients as well as between cisplatin-CR and cisplatin-IR patients. However, these results were not consistent with the in vitro cell experiments. In the present study, knockdown of FBP1 *in vitro* not only attenuated cell proliferation activity but also increased ovarian cell apoptosis induced by cisplatin treatment, indicating that knockdown of FBP1 increases the sensitivity of ovarian cells to cisplatin. However, additional research is required to verify these results.

**Figure 5:**
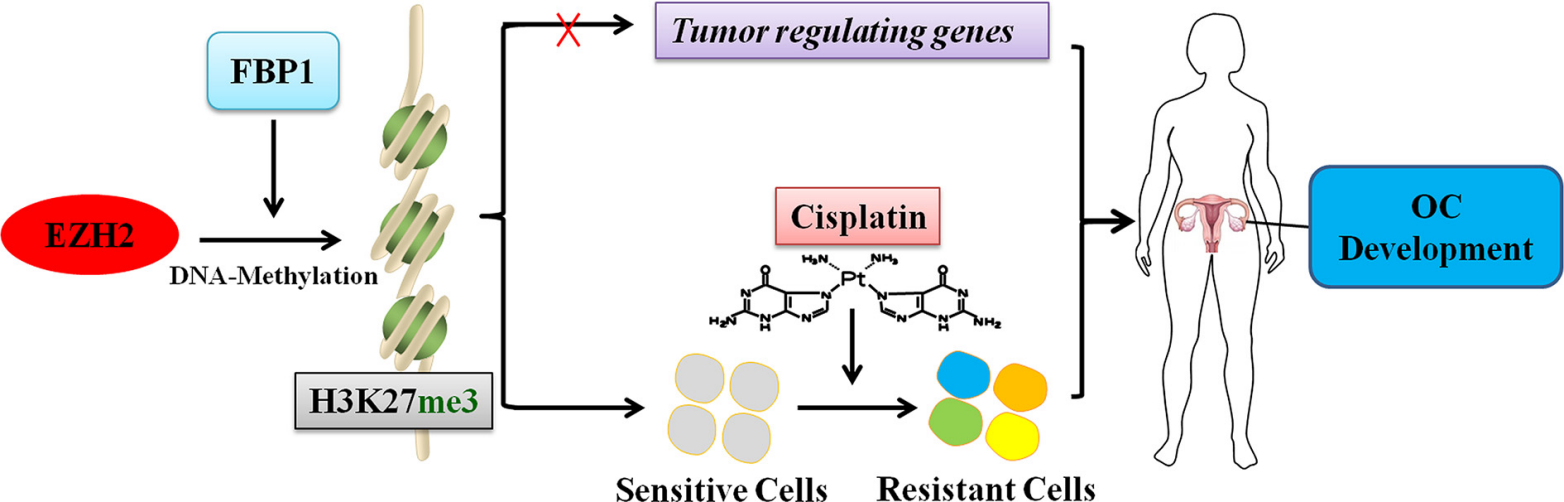
Schematic illustration of FBP1 promoting OC development

To further investigate the role of FBP1 in OC, we analyzed the GSEA dataset and found that high FBP1 expression was enriched in critical biological functions related to tumorigeneses, such as epigenetic regulation, histone methylation and DNA methylation promoted by PRC2. EZH2, a subunit of the PRC2 epigenetic regulator, is involved in tumor progression [32]. In the present study, we demonstrated a positive correlation between FBP1 and EZH2 in ovarian tissues by analyzing TCGA dataset. In addition, we confirmed the internal physical interaction between FBP1 and EZH2 in OC cells. High expression of EZH2 is found in a variety of tumors [64,65], including OC [66]. EZH2 not only promotes the proliferation and metastasis of OC but also plays an important role in drug resistance [44,45,67]. In the present study, the mRNA and protein expression levels were significantly reduced with FBP1 knockdown. H3K27me3, the downstream target of EZH2, was also decreased in FBP1 knockdown OC cells. Furthermore, analysis of clinical ovarian and transplanted animal tumors clearly indicated a significantly positive correlation of FBP1, EZH2 and H3K27me3 expression with OC malignancy. The expression levels of EZH2 and H3K27me3 were significantly higher in FBP1 control tumors than in FBP1 knockdown tumors. These data demonstrated that knockdown of FBP1 decreases the expression of EZH2 and H3K27me3. Thus, we postulated that the knockdown of FBP1 enhances cisplatin sensitivity of OC via down-regulation of the EZH2/H3K27me3 pathway (Figure 5).

In summary, the present study showed that FBP1 expression was closely associated with clinical characteristics of OC. Knockdown of FBP1 attenuated cell proliferation and enhanced cisplatin cytotoxicity potentially by down-regulating EZH2/H3K27me3. These results provide evidence that FBP1 depletion may be a promising intervention for OC treatment.

## Data Availability

The datasets in the manuscript are freely available in the Oncomine dataset (https://www.oncomine.org), the TCGA (https://tcga-data.nci.nih.gov/tcga/), the GEO database (https://www.ncbi.nlm.nih.gov/geo/), and the GSEA (https://www.gsea-msigdb.org/gsea/index.jsp).

## Abbreviations

CIP2A: cancerous inhibitor of protein phosphatase 2A
Co-IP: co-immunoprecipitation
FDR: false discovery rate
GSEA: gene set enrichment analysis
HMGB3: high-mobility group box 3
IHC: immunohistochemical
IOD: integral optical density
NES: normalized enrichment score
